# Implementation and operation of incident learning across a newly‐created health system

**DOI:** 10.1002/acm2.12447

**Published:** 2018-09-17

**Authors:** Leah Schubert, Josh Petit, Yevgeniy Vinogradskiy, Rick Peters, Jack Towery, Bryan Stump, David Westerly, Jane Ridings, Patrick Kneeland, Arthur Liu

**Affiliations:** ^1^ Department of Radiation Oncology University of Colorado School of Medicine Aurora CO USA; ^2^ University of Colorado Health Poudre Valley Hospital Fort Collins CO USA; ^3^ University of Colorado Health Memorial Hospital Colorado Springs CO USA; ^4^ Hospital Medicine Section Division of General Internal Medicine Department of Medicine University of Colorado School of Medicine Aurora CO USA

**Keywords:** incident learning system, quality improvement, safety

## Abstract

**Purpose:**

The purpose of this work is to describe our experience launching an expanded incident learning system for patient safety and quality that takes into account aspects beyond therapeutic dose delivery, specifically imaging/simulation incidents, medical care incidents, and operational issues.

**Methods:**

Our ILS was designed for a newly created health system comprised of a midsized academic hospital and two smaller community hospitals. The main design goal was to create a highly sensitive system to capture as much information throughout the department as possible. Reports were classified according to incidents and near misses involving therapeutic radiation, imaging/simulation, and patient care (not involving radiation), unsafe conditions, operational issues, and accolades/suggestions. Reports were analyzed according to impact on various steps in the process of care. Actions made in response to reports were assessed and characterized by intervention reliability.

**Results:**

A total of 1125 reports were submitted in the first 23 months. For all three departments, therapeutic radiation incidents and near misses consisted of less than one‐third of all reports submitted. For the midsized academic department, operational issues and unsafe conditions comprised the largest percentage of reports (70%). Although the majority of reports impacted steps related to the technical aspects of treatment (simulation, planning, and treatment delivery), 20% impacted other steps such as scheduling or clinic visits. More than 160 actions were performed in response to reports. Of these actions, 63 were quality improvement interventions to improve practices, while 97 were learning actions for raising awareness.

**Conclusions:**

We have developed an ILS that identifies issues related to the entire process of care delivery in radiation oncology, as evidenced by frequent and varied reported events. By identifying a broad spectrum of issues in a department, opportunities for improvement can be identified.

## 1 INTRODUCTION

In radiation oncology, incident learning is increasingly used to improve patient safety and is recommended by numerous organizations.^1^, ^2^ Incident learning systems (ILS) facilitate the reporting of safety events, further analysis for relevant details, and development of interventions to prevent repeat occurrences.^3^ Incidents traditionally refer to events that cause or can potentially cause an adverse effect. In radiation oncology, incident learning has primarily focused on events involving therapeutic doses of radiation. The entire process involves a multidisciplinary team of staff members that must perform several steps in a specific sequence. Thus, an ILS can be used to capture issues throughout the entire process of care, providing more opportunities for improvement.

A number of individual institutions have reported on their experience with incident learning. These publications have primarily analyzed reports within their ILS.^5^, ^6^, ^7^, ^8^, ^9^ Recent studies have indicated early successes with incident learning, such as staff reported safety culture improvements^10^, ^11^ and trending reductions in incident severity levels.^5^ In radiation oncology, however, there are limited reports providing practical details on the clinical implementation and operation of an ILS. This work describes our clinical experience with incident learning that takes into account aspects beyond therapeutic dose delivery, specifically imaging/simulation incidents, medical care incidents, and staff operational issues.

## 2 MATERIALS AND METHODS

Our ILS was designed for a newly created health system comprised of a midsized academic hospital (referred to as Central) and two smaller community hospitals (referred to as North and South). The radiation oncology departments in each hospital were independently operated and staffed. Prior to the implementation of this ILS, each department had its own reporting system. The ILS was implemented first at Central, then expanded to North and South, respectively.

### 2.A Goal‐based design

The initiative to create an ILS at Central was led by a physician and physicist. This ILS served as the program's central source of information to drive improvement efforts. The ILS was designed to achieve the following goals: (a) capture as much information throughout the department as possible, (b) respond swiftly to serious problems, (c) monitor trends and identify problem areas, and (d) allow seamless expansion to accommodate the growing health system.

**Figure 1:**
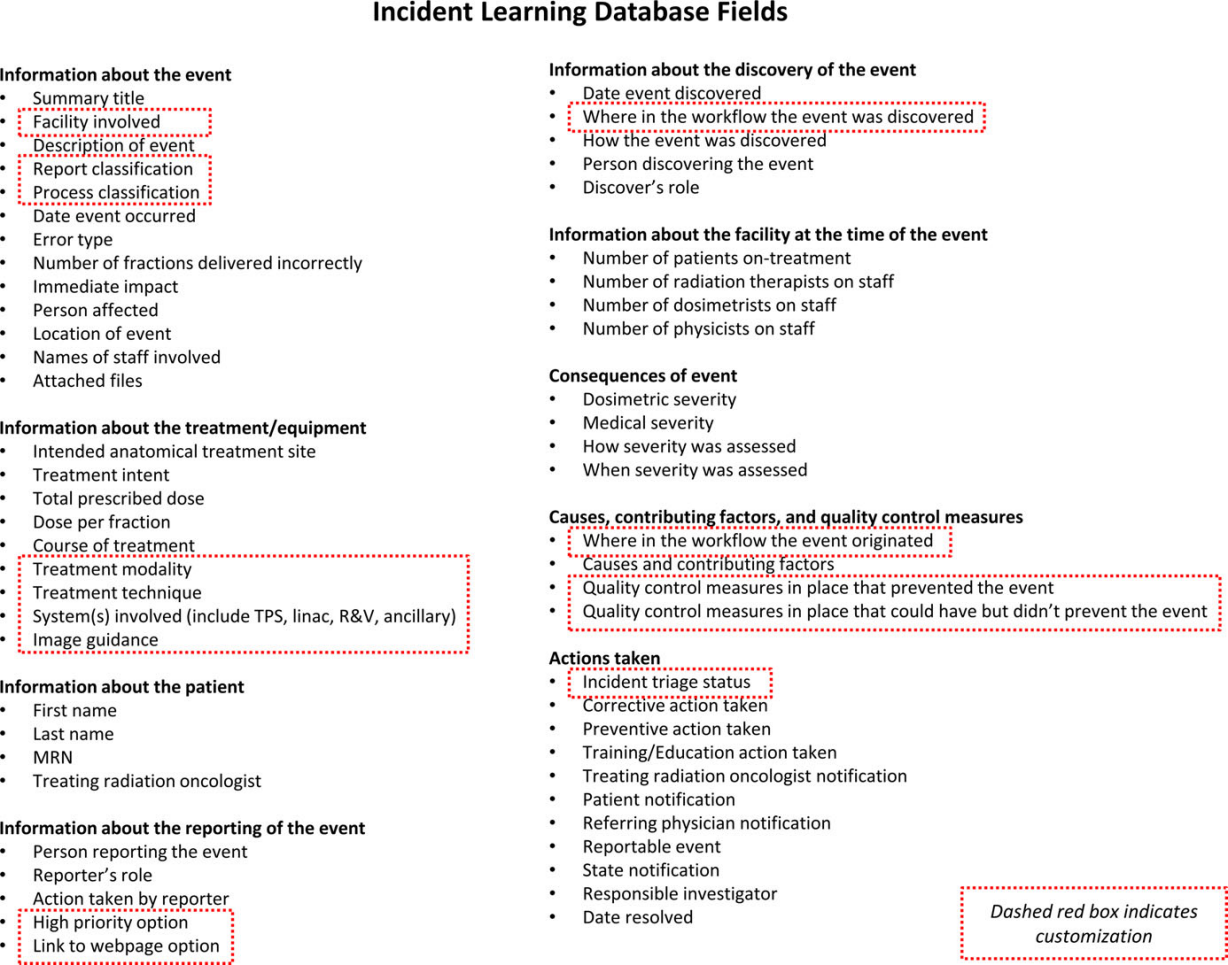
Database fields within the incident learning system designed per AAPM consensus guidelines and further customized (indicated by dashed red boxes).

The ILS was implemented as a voluntary reporting system in which reporting guidelines were intentionally nonprescriptive (i.e., zero threshold reporting). A secure, electronic platform was chosen and maintained by the health system's information technology staff. The platform was available to all radiation oncology staff in the health system. The ILS was initially created with database fields using AAPM taxonomy^3^ and then customized to better reflect our quality and safety needs. Figure**** 1 displays the individual database fields in the ILS, including fields that were customized. For example, the report type classification field in the original AAPM taxonomy document only listed “near miss” or “actual event”, but we wanted to capture additional reports not solely related to patient safety events. This field was subsequently expanded to describe such issues as unsafe conditions, operational issues, as well as positive comments (for staff recognition) and staff suggestions (not necessarily linked to a near miss or actual event). A field for process classification was added, in which field options listed key steps in the process of care within the department (e.g., patient scheduling, insurance authorization, simulation, etc.). This field provided a simple way to track problem areas and identify opportunities for larger‐scale quality improvement projects. The equipment and treatment technique database fields were also customized to be specific to the department. Finally, fields were added to assist in managing responses to incident reports. A field for high priority was added to the report form to allow staff to communicate requests for faster responses. An incident triage status field was added to enable the monitoring and follow‐up of reports. In order to respond swiftly to specific reports, the system automatically sent emails to a quality and safety committee, which provided immediate notification and descriptions of new reports.

### 2.B System launch to facilitate staff reporting

The following sections describe the ILS clinical implementation strategy at Central. This department is within an academic hospital and consists of over 80 employees, a variety of training programs, and uses five linear accelerators plus numerous special procedures to treat over 100 patients a day.

Prior to launch, the concept and goals of our ILS were announced at department‐wide staff meetings, with the department administrator and vice chair relaying the importance of involvement from all staff. Staff were encouraged to report any suboptimal observation, no matter how small. Staff were then trained on how to submit a report. To minimize the fear of potential punishment, reports were treated in a nonpunitive and confidential manner, which was emphasized during the launch meetings.

Efforts were made to minimize reporting barriers. Access to the reporting form was maximized. Desktop shortcuts were placed on work computers throughout the department. To minimize the time spent submitting a report, the reporter's form was made as simple and clear as possible. The reporter's form consisted a free text short title, radial button to select the department, free text description of the event, a patient identifier option, a high priority option, and a save button to submit the report. This simplistic interface was intentionally designed to reduce reporting burden to the department staff, with the understanding that additional information would be collected during follow up investigation.

### 2.C Establishing a method of responding to reports

A committee was formed to lead Central's quality and safety program to respond to reports. The committee consisted of physician, physicist, nurse, therapist, dosimetrist, and administrative staff representatives. Committee members were requested to have open attitudes: not to react to reports in a negative way, but rather to focus on how processes could potentially be improved. The committee collectively had frontline experience with clinical processes, ability to influence change, and a focus on making improvements.

Committee members received immediate emails when a new report was entered into the ILS. Any member could then request follow up investigation to any single high priority report. A high priority report was defined as any actual incident of incorrect therapeutic radiation delivery. Other reports were also considered high priority if they had risk attributes adapted from AAPM guidelines^12^: high severity of impact if the incident had actually occurred, low level of detectability, or high probably of occurrence. If any committee member decided that a report was high priority, then immediate follow‐up was performed.

Follow‐up investigation ranged from an informal interview with the reporter (for minor events) to a more formal root cause analysis (for major events). The methodology of conducting a root cause analysis (RCA) evolved as QI committee members increased their experience and training in RCA. Specifically, the AAPM Incident Learning Workshop and our institution's Institute for Healthcare Quality, Safety, and Efficiency demonstrated many of the methods that are now used in Central's department. A group meeting is now held with all available staff involved in the event, and one member of the QI committee leads the discussion. This QI committee member would not have been involved in the event in order to maximize staff comfort level and minimize any bias during the discussion. The sequence of events leading up to the incident would be reconstructed in order to identify what happened. The individual steps in the sequence of events would be assessed to identify why they occurred, by successively asking the questions “why” until as many as possible causes and contributing factors were identified. Next, the group discussed the list of causes and contributing factors in order to identify (a) root causes and factors that were key contributors to the incident, and (b) additional factors indicating areas of improvement. The group would then brainstorm potential QI interventions to implement. Goals of these QI interventions included preventing the incident from occurring again and/or improving operations. The findings of the RCA were presented to the QI committee. The QI committee then discussed QI interventions to implement, while also continued to monitor implementation progress. Note during the RCA events, the taxonomy in the ILS was not used to classify causes and factors identified as part of the RCA. Rather, these causes and factors were identified on a very fine level of detail specific to the particular incident. This allowed the identification of QI interventions that addressed the incident and specific processes involved.

Regular 1 h our bi‐weekly meetings were established to review reports and to ensure that progress was being made. During these meetings, all new reports were reviewed (on average 5–10 reports per week). If reports were considered high risk, or if the causes indicated that interventions should be implemented, they were selected for follow‐up action.

If a report was selected for follow‐up action, options were brainstormed collectively by the committee. Actions consisted of various QI interventions and learning activities.^3^ QI interventions were defined as actions aiming to improve processes or resources. Learning activities were defined as actions that aimed to raise awareness of the report, for example presentations at staff meetings. The committee decided which options to implement via group consensus. Deciding factors included whether the option addressed a problem's cause or contributing factors, whether the option would be effective, whether the amount of effort needed for clinical implementation was justified, whether the option would cause other problems, and whether staff would likely comply. In order to ensure that progress was being made, the status of ongoing implementation efforts was monitored during the committee meetings.

After the ILS was implemented and tested at Central's radiation oncology department, it was expanded to North and South. Reports for all three sites were stored in the same database, with each department separately accessed and maintained their respective reports. Committee members from each department had the ability to receive email notifications from each other's department.

### 2.D Analysis of reports

**Figure 2:**
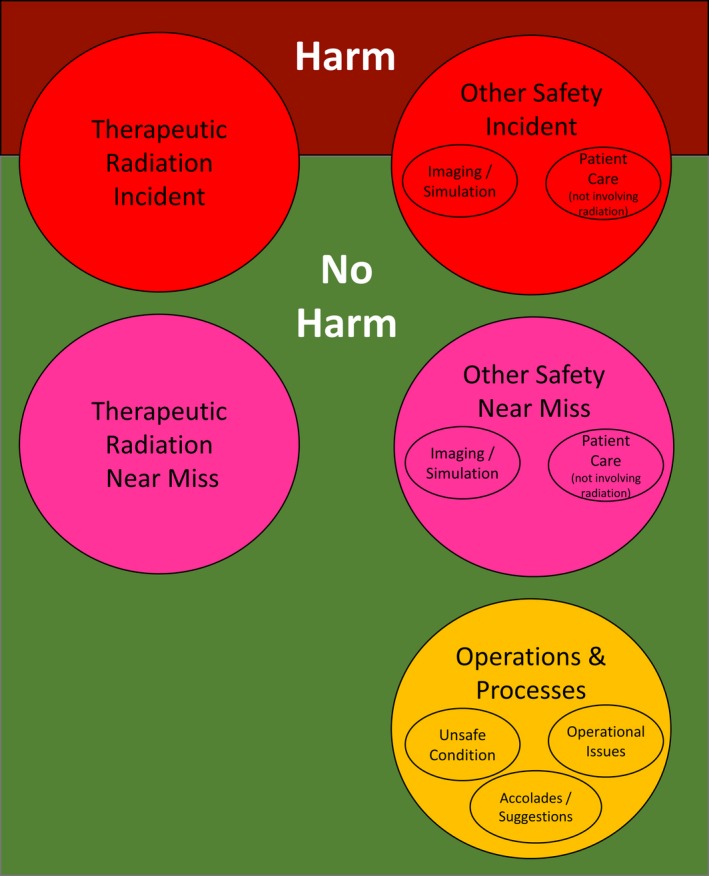
Schematic of classification of report type, adapted from the ROILS definitions. Reports indicated on the left side of the figure (therapeutic radiation incident and near miss, with or without harm) are the types of reports focused on by traditional ILS. Reports on the right side of the figure show reports commonly captured by our expanded ILS.

North's, Central's, and South's reports that were related to therapeutic radiation incidents and near misses were compared with those reports that were not (visually displayed on the left side of Fig.**** 2). The definition of therapeutic radiation incidents was adapted from ROILS^13^ and defined as radiation dose (of therapeutic levels) not delivered as intended, with or without harm. Therapeutic radiation near misses were defined as events that could have directly resulted in a therapeutic radiation incident, but did not.

Central's reports were then analyzed in more detail. These reports were further classified using definitions from ROILS^13^ and adapted to apply to the new types of reports collected in our ILS (visually displayed on the right side of Fig.**** 2). Reports were stratified into other safety incidents (defined as care, not involving therapeutic doses of radiation, that was not delivered as intended), other near misses (defined as events, not involving therapeutic doses of radiation, that could have directly resulted in a deviation of intended delivery of care but did not), and events related to suboptimal operations and processes. Other safety incidents and near misses were substratified into imaging/simulation and patient care not related to radiation. Suboptimal operations and processes were substratified into unsafe conditions (defined as conditions, such as stressors to the process of care, that increased the probability of any safety event,^13^ yet would not have by themselves directly resulted in one) and operational issues (defined as conditions that negatively impacted the staff work environment or efficiencies of departmental processes). Accolades/suggestions consisted of positive accolades (defined as peer‐reported recognitions for jobs well done or exceptional good catches that prevented an error from reaching the patient) and staff suggestions for improvement.

Reports were classified according to steps in the process of care that were impacted. The impacted process steps were defined as any point(s) in the process of care that the problem impeded or negatively affected. The numbers of reports submitted by different staff members were also analyzed.

Actions made in response to reports were assessed. Actions were categorized into learning activities and QI interventions. Of those QI interventions, further categorization was performed according to (a) scale of effort, and (b) effectiveness of intervention. Large‐scale projects involved implementation of more than one QI intervention. Smaller‐scale QI interventions were defined as interventions carried out in response to individual reports (i.e, not associated with a large‐scale project). The effectiveness of each QI intervention was assessed according to level of reliability defined by the Joint Commission^14^ and adapted by Kim, et al.^15^ Most reliable interventions were defined as forcing functions, physical stops preventing incorrect actions, computerized automation, or human‐machine redundancy. Somewhat reliable interventions were checklists, forced pause to recheck details and steps, reminders, standardization of equipment, or planned error‐recovery opportunities. Least reliable interventions were rules, policies, and procedures, or education and training.

## 3 RESULTS

**Figure 3:**
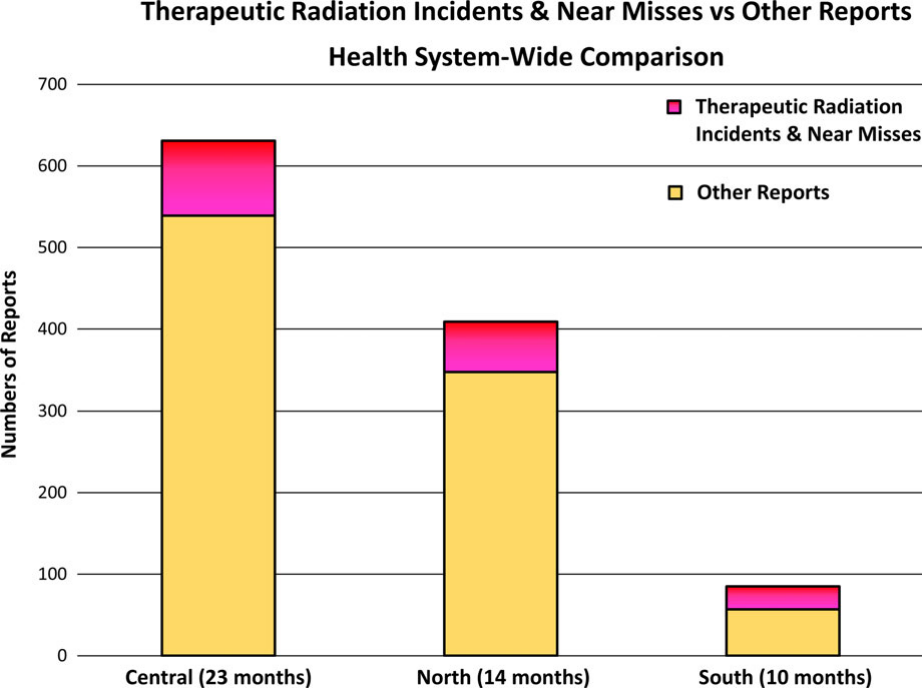
Numbers of therapeutic radiation incidents and near misses for the three radiation oncology departments in the health system. Therapeutic radiation incidents and near misses are shown in gray, while all other reports are shown in red. For all departments, therapeutic radiation incidents and near misses consisted of the minority of total reports.

A total of 1125 reports were submitted across all three departments in the first 23 months. Totals of 631, 409, and 85 reports were submitted for Central, North, and South during a 23, 14, and 10‐month time period, respectively. For all three departments, therapeutic radiation incidents and near misses consisted of the minority of all reports submitted (15% for Central, 15% for North, and 33% for South) (Fig. 3).

**Figure 4:**
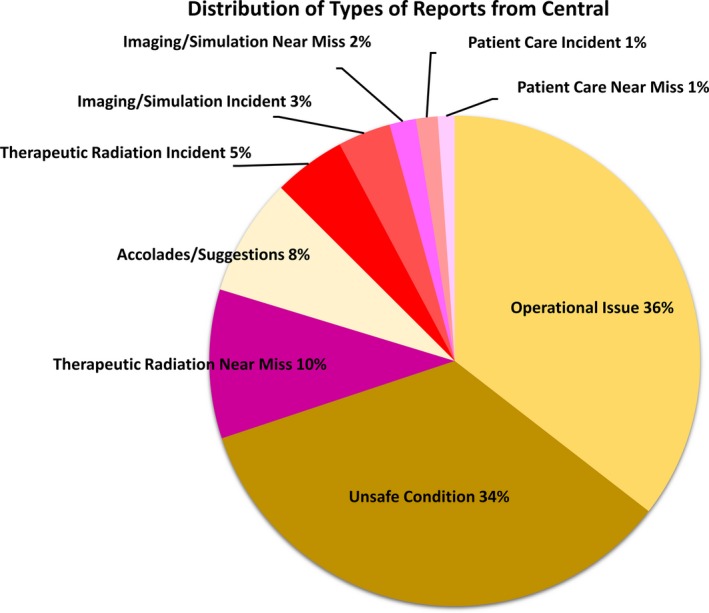
Distribution of reports from Central classified according to report type: incidents shown shades of red, near misses shown in shades of purple, and operations and processes shown in shades of yellow.

Figure 4 shows Central's reports further substratified. Operational issues comprised the largest percentage of reports (36%), followed by unsafe conditions (34%). An example of an operational issue involved on‐treatment clinic visit scheduling. An example of an unsafe condition was treatment start being rushed due to delays in planning.

**Figure 5:**
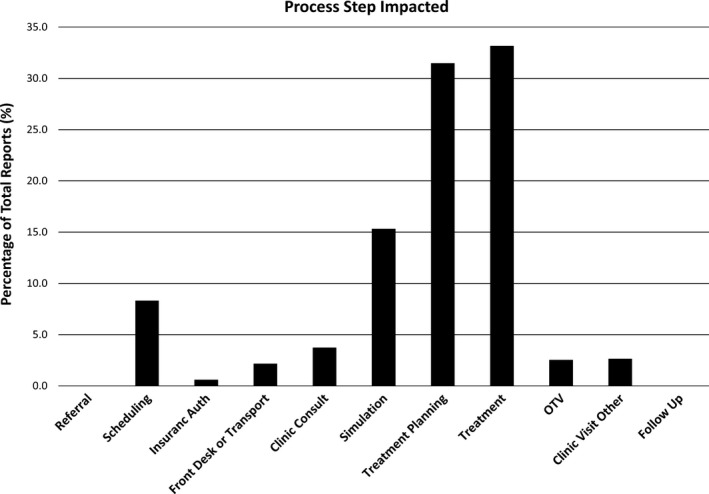
Distribution of reports (percentage of the total) impacting different steps in the entire process of care in a radiation oncology clinic. Impact is defined as any point(s) in the process of care that the problem impeded or negatively affected.

**Figure 6:**
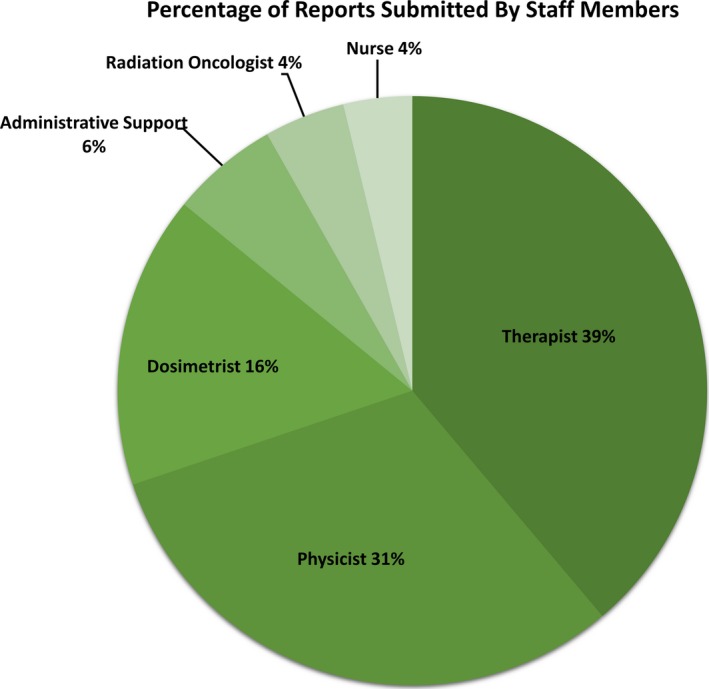
Distribution of reports submitted by staff members from various staffing groups throughout the department, indicated by varying shades of green.

Reports impacting different steps in the process of care are shown in Fig.**** 5 **.** While the majority of reports impacted steps related to the technical aspects of treatment (simulation, planning, and treatment delivery), 20% impacted other steps such as scheduling or clinic visits. Reporters consisted of staff members from various staffing groups throughout the department. Figure**** 6 displays the percentage of reports submitted by the staffing groups. Therapists submitted the highest percentage of total reports, while nurses and radiation oncologists submitted the lowest percentage of reports.

More than 160 actions were performed at Central in response to incident reports submitted over the 23 month period. Of these actions, 63 were QI interventions implemented to improve practices or resources, while 97 were learning actions to raise awareness. Of the 63 QI interventions, 18 were in response to reports not related to therapeutic radiation incidents or near misses. An example of an intervention not related to therapeutic radiation was the elimination of myelogram procedures performed within the department, which had caused staffing and equipment coordination difficulties.

Seven large‐scale projects were enacted, resulting in 46 QI interventions. Large‐scale projects consisted of improvements to the (a) high dose rate (HDR) brachytherapy program,^16^ (b) total body irradiation (TBI) program, (c) use of bolus, (d) clinical electron setups, (e) risks of physical collisions throughout the department, (f) hands‐on department‐wide emergency training, and (g) use of optional software options to enhance safety.

**Figure 7:**
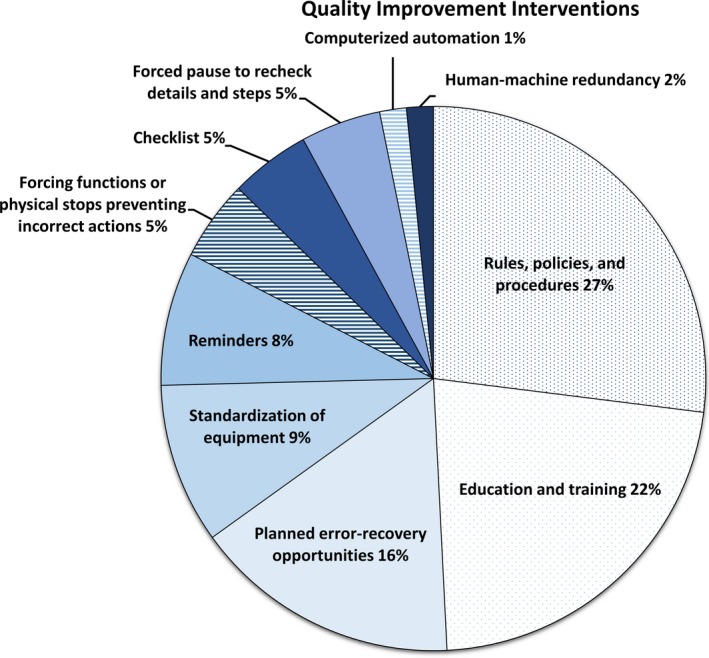
Classification of quality improvement interventions implemented in response to reports by type and reliability. The horizontal lines indicate high reliable interventions. The solid sections indicate somewhat reliable interventions. The dotted sections indicate least reliable intervention.

We implemented 17 smaller‐scale QI interventions in response to individual reports, not associated with a large‐scale project. An example of a smaller‐scale QI intervention was improving processes at simulation to prevent treatment‐site laterality errors. Figure**** 7 categorizes all QI interventions according to reliability. Interventions were commonly least reliable (49%), followed by somewhat reliable (43%), and most reliable (8%).

Numerous steps were taken to establish a safety culture when responding to reports. When reports were presented at staff meetings, names of those involved were not mentioned and emphasis was made on the improvements implemented in response. Potential incidents, many of which were caught during standard quality assurance checks, were often presented as “good catches.” Awards were given out when a staff member made an exceptional catch. Staff members were encouraged to enter reports of a positive nature into the ILS, specifically if they noted peers performing either exceptionally well at their jobs or to promote safety. These peers were recognized during staff meetings.

## 4 DISCUSSION

Our ILS was successfully implemented across three independently operating radiation oncology departments, as indicated by over 1000 reports submitted within a 23‐month period. The majority of reports were not related to therapeutic radiation incidents nor near misses. This trend was observed in all three departments. For Central, the most common reports were operational issues and unsafe conditions. The prevalence of these reports is likely due to our implementation strategy that emphasized the reporting of any suboptimal workflow. Others have reported numerous unsafe conditions or operational issues in their ILS. In the first year of the ROILS experience, unsafe conditions made up 23% of their submitted reports.^17^ It has been argued that latent failures of a system result in incidents,^4^ which is one reason why near misses are valuable learning experiences. Identifying additional underlying operational stressors could provide more opportunities for improvement. An ILS broadens the role of quality improvement efforts to include the entire department, not just those processes that are technical in nature. All members of the department, including administrative and medical staff, have the potential to benefit from an ILS.

There is limited published work that emphasizes practical actions made in response to reports.^15^, ^18^ Kim, et al. recently published their responses to their incident reports related to TBI.^15^ We used their classification schema to assess the effectiveness of our responses and found similarities. In particular, only a minority of implemented interventions could be classified as most effective. As Kim, et al. has discussed, more reliable interventions typically require specific skills or resources.^15^ It may simply be easier to implement less reliable interventions if one has limited time or resources. Experience with incident learning could be another factor. Identifying interventions depends on the background, experience level and attitudes of staff. It would be interesting to see how the effectiveness of our responses changes over time as our experience with incident learning evolves. Additionally, more reliable interventions could be identified by broadening the idea pool during brainstorming sessions. In one of our large‐scale projects, North, Central, and South worked together to improve our use of bolus by sharing best practices.

The Radiation Oncology Incident Learning System (ROILS) is uniquely positioned to share ideas on a national basis. ROILS is the national incident learning system,^17^ and it provides powerful opportunities to study safety events across the entire nation and disseminate widespread learning to the entire radiation oncology community. Each individual clinic can use ROILS internally for their own ILS, which is beneficial because it does not require the resources for the technical development and maintenance of the software. We started developing our ILS in 2013, which was before ROILS was available. Rather than immediately switching systems, we decided to spend those subsequent years improving our methods to respond to reports, implementing QI interventions, and engaging staff. We are in the process of applying to ROILS due to its advantages on a national scale. The practical strategies that we identified can be easily applied other clinics considering joining ROILS.

A thoughtfully implemented ILS is necessary for successful adoption. In our experience, several strategies contributed to the successful implementation of an expanded ILS. Support from leadership, specifically department administration, physician, and physicist champions, helped to drive involvement from the entire department. Minimizing the burden for staff to enter a report was critical. Another key factor was the makeup of the QI committee. Members had a combination of front‐line responsibilities and supervisory roles. This allowed meaningful brainstorming of interventions along with a higher likelihood that staff would comply with resulting changes. The attitudes of committee members were also important. Members focused on improving the department, as opposed to taking a punitive view.

Sustaining an active ILS requires additional resources.^19^ The main practical challenges we experienced were the extra time and effort needed to respond to reports and clinically implement interventions. A challenge we discovered early on was realizing that we could not respond to all reports, which has also been acknowledged by others.^15^ Because patient safety was the most important goal of our ILS, the less frequent incidents and serious near misses took priority over the more common operational issues. Additional challenges we continue to experience are communicating back to staff and maintaining positive engagement. Prior to implementing the ILS, only the physics group discussed therapeutic radiation incidents. We have since established formal communication opportunities by adding standing agenda items to numerous department meetings. In the first 2 years, we discussed reports at various meetings 97 times in order to raise awareness of various safety events and operational issues. Our ILS has opened up communication to the entire department about such topics.

This work has limitations. The strategies that we used to implement incident learning worked for our system. While we hope this description of our experience will inform others interested in establishing incident learning, the success of such strategies will depend on the people, culture, and available resources of the specific department. Additionally, incident learning has inherent limitations. One weakness of a voluntary ILS is underreporting, resulting in potential reporting bias. In our analysis, therapists, physicists, and dosimetrists submitted the most reports, which indicates a potential reporting bias toward issues more technical in nature. Finally, it is difficult to tell from the first 2 years of our experience whether we have actually improved safety and quality. As has been discussed,^5^ a true metric for measuring safety can be challenging to identify. The ultimate effectiveness of incident learning depends on the effectiveness of and compliance to QI interventions implemented in response to reports.

## 5 CONCLUSIONS

We have successfully implemented an ILS that identifies issues related to the entire process of care in radiation oncology, as evidenced by frequent and varied reported events. By identifying a broad spectrum of issues in a department, opportunities for improvement were identified. We have also expanded to multiple, independently operating departments, which provide further opportunities for improvement in quality and safety.

## CONFLICT OF INTEREST

Conflicts of interest do not exist.
